# Actin Polymerization Negatively Regulates p53 Function by Impairing Its Nuclear Import in Response to DNA Damage

**DOI:** 10.1371/journal.pone.0060179

**Published:** 2013-04-02

**Authors:** Ling Wang, Min Wang, Shuyan Wang, Tianyang Qi, Lijing Guo, Jinjiao Li, Wenjing Qi, Khamal Kwesi Ampah, Xueqing Ba, Xianlu Zeng

**Affiliations:** Key Laboratory of Molecular Epigenetics of MOE and the Institute of Genetics and Cytology, Northeast Normal University, Changchun, Jilin, China; Institut de Génétique et Développement de Rennes, France

## Abstract

Actin, one of the most evolutionarily conservative proteins in eukaryotes, is distributed both in the cytoplasm and the nucleus, and its dynamics plays important roles in numerous cellular processes. Previous evidence has shown that actin interacts with p53 and this interaction increases in the process of p53 responding to DNA damage, but the physiological significance of their interaction remains elusive. Here, we show that DNA damage induces both actin polymerization and p53 accumulation. To further understand the implication of actin polymerization in p53 function, cells were treated with actin aggregation agent. We find that the protein level of p53 decrease. The change in p53 is a consequence of the polymeric actin anchoring p53 in the cytoplasm, thus impairing p53 nuclear import. Analysis of phosphorylation and ubiquitination of p53 reveals that actin polymerization promotes the p53 phosphorylation at Ser315 and reduces the stabilization of p53 by recruiting Aurora kinase A. Taken together, our results suggest that the actin polymerization serves as a negative modulator leading to the impairment of nuclear import and destabilization of p53. On the basis of our results, we propose that actin polymerization might be a factor participating in the process of orchestrating p53 function in response to DNA damage.

## Introduction

DNA damage contributes to the development of many human cancers [1]. Cells respond to DNA damage by inhibiting DNA synthesis, allowing them to avoid progressive increases in genomic changes and neoplastic transformation [2], [3]. p53 plays an important role in the inhibition of DNA synthesis by activating cell cycle arrest related genes [4]–[7]. In normal condition, inactive wild-type p53 is predominantly localized in the cytoplasm and enters the nucleus during the G1-S transition in cells [8]. In response to DNA damage, p53 rapidly accumulates in the nucleus, where it functions as a transcriptional factor to modulate the expression of cell-cycle- and/or apoptosis -related genes [9]. The classical view of p53 activation includes three steps: p53 stabilization, DNA binding, and transcriptional activation [10]. A key step in stabilization of p53 is its transport into the nucleus. Nuclear import of p53 in response to stress may serve as a rapid mechanism of p53 stabilization by removing it from the cytoplasm [11]–[13]. Several proteins have been shown to be involved in regulating p53 nuclear transport. MDM2 (the Murine Double Minute 2 oncogene) regulates p53 by promoting its nuclear export and degradation through the ubiquitin-proteasome pathway [14]. Inhibition of MDM2 function by ARF (Alternative Reading Frame) tumor suppressor protein or by an antisense oligonucleotide resulted in a translocation of p53 from the cytoplasm to the nucleus [15]. Mot2 (mortalin 2), an hsp70 (the 70 kilodalton heat shock proteins) family member, interacts with p53 and inhibits its nuclear import [16]. These proteins regulate p53 cellular localization positively or negatively, and orchestrate the choice of p53 between different subsets of cell cycle arrest genes or apoptosis genes in response to various level of DNA damage [17].

Actin plays essential roles in numerous cellular processes such as establishing and maintaining cellular polarity, driving cell shape changes, as well as cell motility, adhesion, cytokinesis, endocytosis and intracellular trafficking [18]–[21]. Previous studies have shown that actin is involved in the process of p53 responding to DNA damage. First, cytoskeletal actin filaments are found closely associating with p53 at the stage of its initial increase in cells in a calcium ion-dependent manner [22]. This is followed by the binding of p53 and F-actin being modulated by the presence of DNA damage [23]. But whether actin participates in the modulation of p53 function in response to DNA damage as well as the exact mechanisms remain unclear.

In this study, we showed that actin polymerization increased along with p53 cellular accumulation in response to ETO-induced DNA damage. The polymerization of actin impaired the nuclear import of p53 by anchoring p53 on the polymeric actin in the cytoplasm. Furthermore, the stabilization of p53 in the cytoplasm decreased due to p53 phosphorylation at Ser315, which was enhanced by Aurora kinase A. Henceforth, the opportunity of the cytoplasmic p53 interacting with Aurora kinase A increased as a result of p53 interacting with polymeric actin. Therefore, our study provides convincing evidence that the interaction of actin and p53 is modulated by actin polymerization in response to DNA damage, and p53 nuclear import is impaired due to the formation of polymeric actin in the cytoplasm. We hypothesized that actin polymerization is important for orchestrating p53 function in response to DNA damage by regulating p53 cellular localization and stabilization.

## Materials and Methods

### Plasmids and siRNA

YFP-tagged p53 expression plasmid and HA-tagged actin expression plasmid were contrusted by PCR and introduced into the pYFP vector and pcDNA3.1 vector, respectively. The sequence of cofilin siRNA [24] was: AAGGUGUUCAAUGACAUGAAA; Aurora kinase A siRNA [25] was: AUGCCCUGUCUUACUGUCAUU; actin siRNA [26] were: UUGGCGCUUUUGACUCAGGA and UGUAAGGUAAGGUGUGCACU.

### Primers for Real Time PCR

The following pairs of primers were used for examing the expression of human p53, GAPDH, p21 and β-actin. p53 forward: 5′-ACCTGGAGTCTTCCAGTGTGAT-3′ and reverse: 5′-AGTCACAGCACATGACGGAG-3′; GAPDH forward: 5′-AACGGATTTGGTCGTATTGGG-3′ and reverse: 5′-CCTGGAAGATGGTGATGGGAT-3′; p21 forward: 5′-GGGATGAGTTGGGAGGAG-3′ and reverse: 5′-TGAGACTAAGGCAGAAGATGTA-3′; β-actin forward: 5′-CACCAACTGGGACGACAT-3′ and reverse: 5′- AGGCGTACAGGGATAGCA-3′. The results were calculated using 2^−ΔΔCt^ method.

### Antibodies and Reagents

Rabbit antibodies against HA tag (Y-11), p53 (FL-393), p53 (DO-1) and p21 (C-19) were from Santa Cruz Biotechnology. Rabbit antibodies against phospho-p53 (Ser315) (2528), phospho-p53 (Ser392) (9281) and Aurora kinase A (4718) were from Cell Signaling Technology, USA. Mouse anti-β-actin antibody (A5441) and Rabbit anti-GAPDH antibody (G9545) were from Sigma, USA. Rabbit anti-cofilin antibody (ab42824) and Rabbit anti-Oct-1 antibody (ab15112) were from Abcam, USA. Mouse anti-γH2AX antibody (05–636) was from Upstate, USA. Etoposide (ETO), a DNA damage inducer used in the present study, was from Sigma. MG-132 was from Sigma. Alexa Fluor 568 phalloidin (A-12380) was from Invitrogen, USA.

### Cell Culture and Transfection

U2OS cells (American Type Culture Collection) was maintained in DMEM supplemented with 10% fetal calf serum, 100 units/ml penicillin and 100 µg/ml streptomycin. The cells were grown at 37°C in the presence of 5% CO_2_. U2OS cells were transfected with plasmids or siRNA using Fugene HD (Roche) according to the manufacturer’s protocol.

### Whole Cell Protein Extraction and Nuclear/Cytoplasmic Extraction

Whole cell protein was extracted with RIPA lysis buffer as described previously [27]. The lysate was centrifuged at 10 000 g for 30 min, and the resultant supernatant was harvested as whole cell extracts. Nuclear extraction and cytoplasmic extraction were prepared by using CelLytic™ NuCLEAR™ Extraction Kit (Sigma) following the manufacturer’s instruction.

### Immunoprecipitation and Western Blotting

Immunoprecipitation was carried out using the whole cell extracts incubated with appropriate antibodies at 4°C for 3 h. Samples were then incubated for another 3 h with Protein A/G–Sepharose. After washing the samples with RIPA lysis buffer, the immunoprecipitates were resolved on SDS-PAGE, electroblotted on to nitrocellulose membranes (Millipore) and probed with antibodies as indicated. Chemiluminescent detection was performed by using ECL plus reagents (Promega).

### GST Pulldown Assays

GST-fused proteins were expressed in *Escherichia coli* BL21 by induction with 1 mM IPTG (isopropyl *β*-Dthiogalactoside) at 37°C for 3 h. The cells were harvested and protein purification was performed as described previously [28]. GST and GST-fused proteins immobilized on 40 µl of glutathione Sepharose 4B were incubated with whole cell extracts on a rotator at 4°C for 3 h. After being washed four times with NP40 lysis buffer [50 mM Tris (pH7.4),150 mM NaCl,1% NP-40, 1 mM PMSF and 10 mg/ml aprotinin], the bound proteins were analyzed by western blotting.

### Confocal Microscopy

Cells were washed with PBS, fixed in 4% (w/v) paraformaldehyde in PBS for 20 min and then permeabilized with 0.2% Triton X-100 in PBS for 5 min. The fixed cells were stained with DAPI for DNA and Alexa Fluor 568 phalloidin for F-actin. Nonspecific binding was prevented by incubation of the permeabilized cells in 10% (v/v) bovine serum or 1% BSA in PBS for 30 min prior to fluorescent staining. Cells were then observed using a laser-scanning confocal microscope (FluoView FV1000; Olympus) equipped with a 60 × oil-immersion objective lens. Fluorescent images were collected using Olympus FV10-ASW 1.7 software. The colocalization and distribution of p53 were analyzed with Image J software.

### Quantitative Analyses of the Cellular Intensity for F-actin

Cells were seeded in 96-well plates at 7 000 cells/well. After 24 h, cells were treated with ETO for the indicated time intervals. Cells were stained with TRITC-phalloidin and DAPI for 60 min. Fluorescence intensities were measured with a fluorescence microplate reader (TRITC excitation/emission: 550/620 nm; DAPI excitation/emission: 358/461 nm) (Gemimi EM, Molecular Devices, USA). Background was defined as the fluorescence level in the wells with no cells, and phalloidin values were normalized to DAPI values that were taken as quantification of cell number.

### Ubiquitination Assay

Cells were transfected with HA-actin plasmid or pcDNA plasmid as control. At 24 h post transfection, cells were treated with or without ETO for 12 h, followed by the treatment of 20 µM MG-132 for 6 h. Whole cell extracts were immunoprecipitated with anti-p53 (DO-1) antibody and analyzed by western blotting with anti-p53 (FL-393) antibody [29].

## Results

### Actin Polymerization Increases in Response to DNA Damage

**Figure 1:**
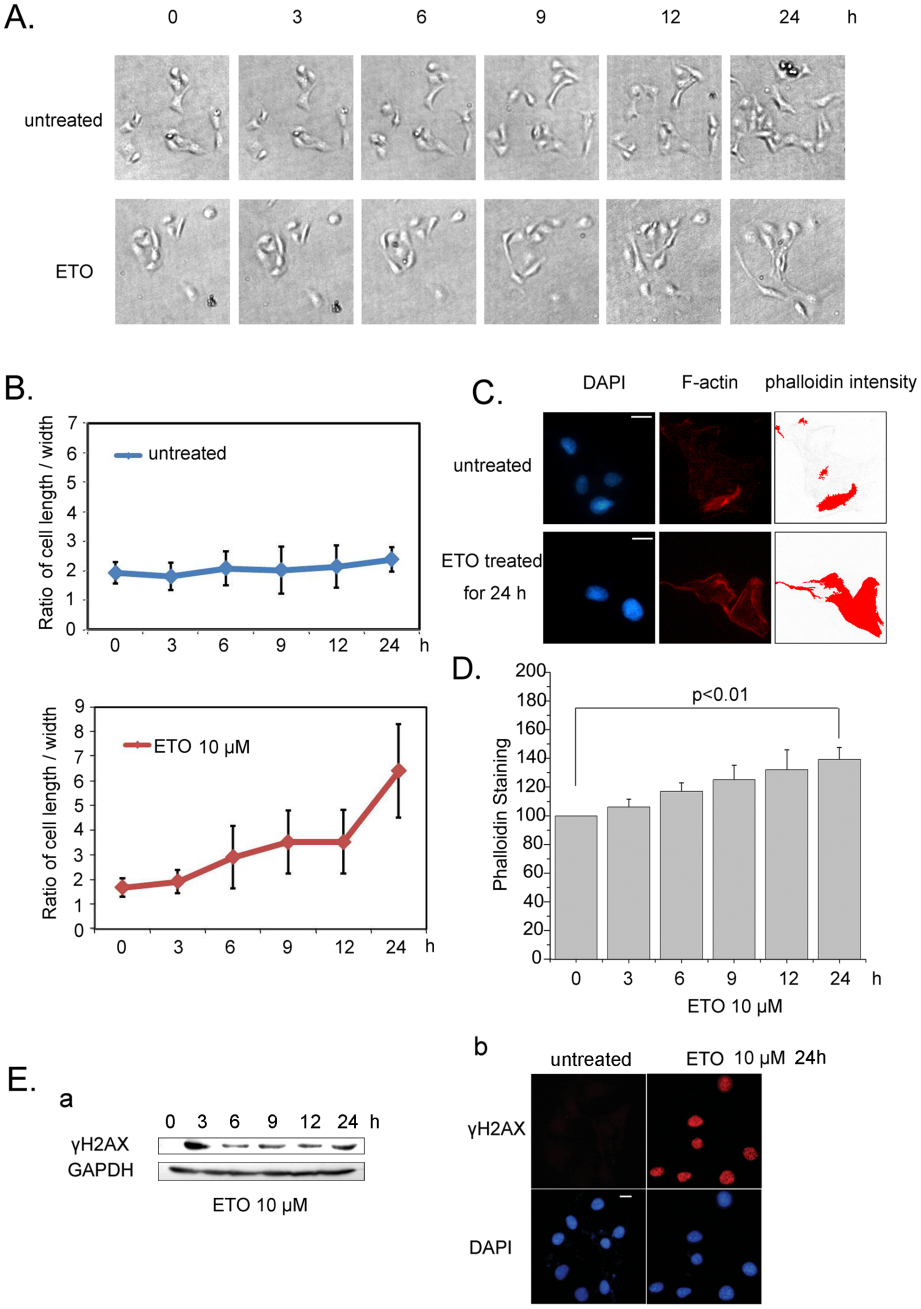
Actin polymerization increases in response to DNA damage. *A.* U2OS cells were treated with ETO (10 µM) or untreated as control for 24 h, and images were captured at the indicated time points. *B.* The cell length and width were analyzed with Image J software in ≥100 cells per condition. *C*. U2OS cells were treated with ETO (10 µM) or untreated as control for 24 h, the intensity of phalloidin was measured with Image Pro Plus software. Scale bar, 10 µm. *D.* U2OS cells were treated with ETO (10 µM) or untreated as control for 24 h, and fluorescence assays were performed with a fluorescence microplate reader to measure cellular F-actin levels (phalloidin intensity/DAPI intensity). *E.* Cells were treated with ETO (10 µM) at indicated time points, and then whole cell extracts were analyzed by western blotting using anti-γH2AX antibody (a). U2OS cells were treated with ETO (10 µM) or untreated as control for 24 h. Then, immunofluorescence was performed to detect the signal of γH2AX (b). Scale bar, 10 µm. All Statistical differences were determined by One-way ANOVA. Results are presented as means ± SD of values from three independent experiments. ETO, etoposide.

Actin interacts with p53, and the interaction is enhanced when DNA damage happens [23], but the elaborate dynamics of actin respond to DNA damage and its physiological significance remain elusive. To explore the dynamic changes of actin upon DNA damage, we first examined the morphology of cells treated with etoposide (ETO), a genetoxic agent that enhances the cleavage of DNA double-strand [30]. As shown in Fig. 1*A*, while the untreated cells retained a normal shape within 24 h (upper panel), the cells with ETO treatment became longer and thinner (lower panel). Then the statistics of cell length over width was performed. As shown in Fig. 1*B*, the tendency of length/width in untreated cells kept smoothly rising. In contrast, the ratio of length/width showed a rapid increase in ETO treated cells.

To confirm the alternation of F-actin, TRITC-phalloidin was used to stain the actin fibers before and after ETO treatment. As shown in Fig. 1*C*, phalloidin intensity was analyzed with Image-Pro Plus, and the intensity in ETO treated cells was obviously stronger than untreated cells. The cellular intensity of phalloidin was measured with fluorescence microplate reader. In the cells treated with ETO, F-actin exhibited a slow increase up to 140% at time of 24 h post-treatment (Fig. 1*D*), which was consistent with previous observations [31]. The level of phosphorylated H2AX- γH2AX, a sign of double-strand cleavage of DNA [30], was measured to confirm the efficiency of ETO treatment inducing DNA damage by western blotting (Fig. 1*E* a). While no signal of γH2AX was detected in control cells, the fluorescence stained for γH2AX could be observed obviously in the cells treated with ETO for 24 h (Fig. 1*E* b). Taken together, these data demonstrate that actin polymerization responds to DNA damage.

### Actin Polymerization Modulates p53 Cellular Accumulation

**Figure 2:**
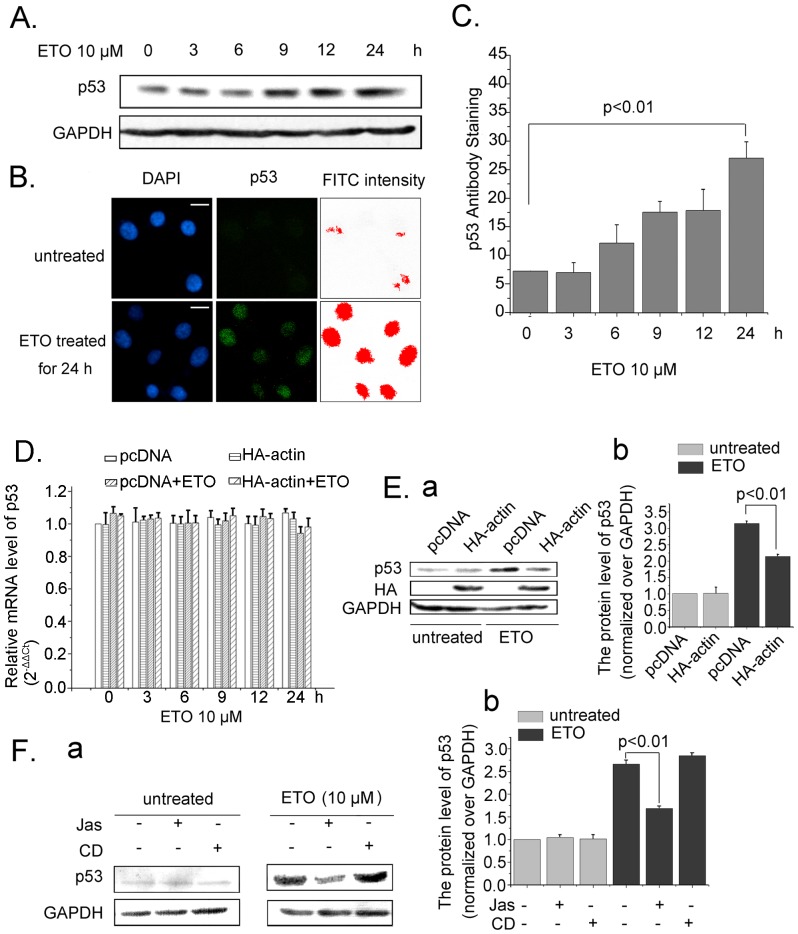
Actin polymerization modulates p53 cellular accumulation. *A*. U2OS cells were treated with ETO (10 µM) or untreated as control for 24 h, cells were harvested and the whole cell proteins were extracted for western blotting to measure p53 protein levels. *B.* U2OS cells were treated with ETO (10 µM) or untreated as control for 24 h, the intensity of FITC staining p53 was measured with Image Pro Plus software. Scale bar, 10 µm. *C*. U2OS cells were treated with ETO (10 µM) or untreated as control for 24 h, and fluorescence assays were performed with a fluorescence microplate reader to measure cellular p53 levels (FITC intensity/DAPI intensity). *D*. U2OS cells were transfected with HA-actin or pcDNA, then treated with ETO (10 µM) or untreated as control at indicated time points. The cells were harvested, RNA extraction and Real Time-PCR were carried out. The mRNA content of p53 was normalized to that of GAPDH and the normal cells’ mRNA level was valued as 1. Data (mean±SD) were from three independent experiments. *E*. U2OS cells transfected with HA-actin were treated with ETO (10 µM) or untreated as control for 12 h. Whole cell proteins were extracted and western blotting was performed. *F*. U2OS cells were treated with Jas (50 nM) or CD (0.01 µg/ml) for 2 h to change actin dynamics, then treated with ETO or not. Cells were harvested, and whole cell proteins were extracted for western blotting to detect p53 protein levels in different conditions (a). Results were analyzed with Image J software (b). All Statistical differences were determined by One-way ANOVA. Jas, Jasplakinolide; CD, Cytochalasin D.

The mRNA and protein levels of p53 were also detected along with ETO treatment. The p53 protein accumulated in cells obviously (Fig. 2*A*). Then, immunofluoresence staining of p53 was performed. FITC intensity assay of p53 confirmed the increase of p53 protein with ETO treatment for 24 h (Fig. 2*B*). As shown in Fig. 2*C*, cellular p53 increase persistently with increasing time intervals of ETO treatment.

Since actin polymerization and p53 protein respond to DNA damage, and actin interact with p53 in cells, we questioned whether actin polymerization is implicated in regulating p53 function. When DNA damage occurs, p53 is accumulated and enters into the nucleus, resulting in cell cycle arrest or apoptosis [32], [33]. In the present study, HA-actin plasmid was used to increase actin protein level in cells. Real Time-PCR experiments demonstrated that p53 mRNA exhibited no change in response to ETO treatment at different time points (Fig. 2*D*). Without effect on untreated cells, transfection of HA-actin diminished the increase of p53 protein in the ETO-treated cells (Fig. 2*E* a), and the change was statistically significant (Fig. 2 *E* b).

To identify whether actin polymerization affects p53 cellular accumulation, cells were treated with Jasplakinolide (Jas), which induces polymerization of monomeric actin into amorphous masses [34], or Cytochalasin D (CD), which inhibits association and dissociation of actin monomers at the barbed end [35]. Then the cells were treated with or without ETO for 12 h, protein extraction and western blotting analysis were performed. As shown in Fig. 2*F* a, ETO treatment resulted in p53 accumulation. However, Jas treatment hampered p53 accumulation induced by ETO, whereas CD treatment had no effect. The difference in p53 protein level between cells treated with ETO and Jas plus ETO was significant (Fig. 2 *F* b). All the above data suggest that actin polymerization modulates p53 cellular accumulation.

### p53 Binds to Polymeric Actin in the Cytoplasm

**Figure 3:**
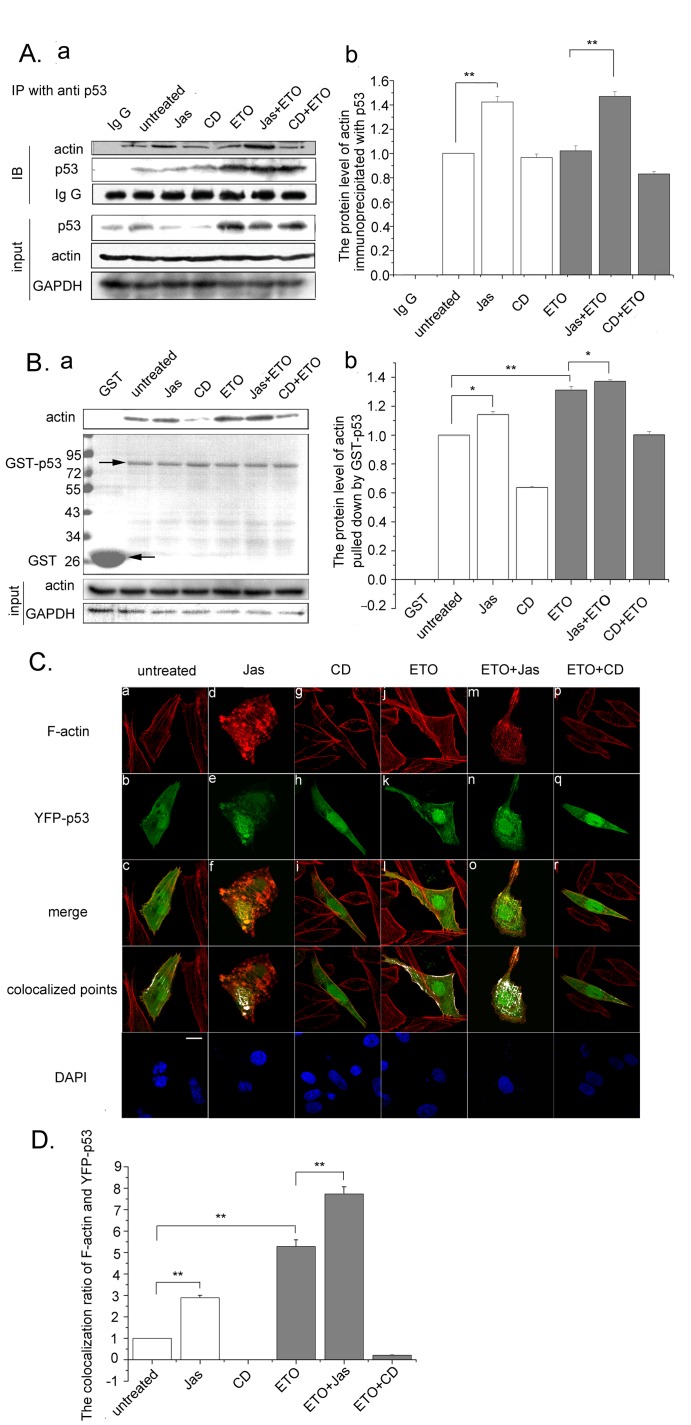
p53 binds to polymeric actin in the cytoplasm. *A.* U2OS cells were treated with Jas (50 nM) or CD (0.01 µg/ml) for 2 h and followed with ETO (10 µM) treatment for another 12 h. The cells were then harvested and subjected to immunoprecipitations using anti-p53 antibody and analyzed by western blotting (a). Western blottings of immunoprecipitated actin were analyzed using Image J software. Results are presented as means ± SD of values from three independent experiments (b). *B.* U2OS cells were treated with Jas (50 nM) or CD (0.01 µg/ml) for 2 h and treated with or without ETO (10 µM) for another 12 h. The cells were then harvested and the whole cell extracts were incubated with GST or recombinant GST-p53. The bound proteins were analyzed by western blotting with anti-actin antibody (top panel). GST and GST–p53 were stained with Coomassie Blue (middle panel). Arrows show the position of GST and GST–p53. Whole cell extracts were immunoblotted with antibody against GAPDH to confirm equal loading (bottom panel) (a). Western blottings of actin were analyzed using Image J software. Results are presented as means ± SD of values from three independent experiments (b).*C*.U2OS cells were treated with Jas (50 nM) or CD (0.01 µg/ml) for 2 h before YFP-p53 transfection. Twenty-four hours after YFP-p53 transfection, cells were treated with ETO (10 µM) for another 12 h or untreated as control and analyzed by confocalmicroscopy (a). Scale bar, 10 µm. The colocalization ratio of F-actin and YFP-p53 of more than 100 cells were calculated using Image J software. Results are presented as means ± SD of values from three independent experiments (b). All Statistical differences were determined by One-way ANOVA. *, P<0.05; **, P<0.01.

To investigate how actin polymerization affects p53 accumulation, immunoprecipitation assay was performed. Actin was found to be co-immunoprecipitated with p53, suggesting that p53 interacts with actin physically (Fig. 3*A* a). Jas treatment resulted in a 1.4-fold of actin amount in p53 immunoprecipitates in the ETO treated cells, CD treatment had no effect on actin binding to p53 (Fig. 3*A* *,* a and b). The results hint an affinity of p53 with polymeric actin.

To confirm the affinity of polymeric actin and p53, a GST pulldown assay was performed. GST and GST-fused p53 were incubated with whole cell extracts. The bound proteins were analyzed by western blotting with an antibody against actin. As shown in Fig. 3*B*, p53 bound strongly to actin in untreated cells. While Jas treatment enhanced the quantity of actin pulled down by GST-p53 (Fig. 3*B* *,* a and b), CD treatment resulted in less binding of actin to GST-p53 than that shown in untreated cells. ETO and ETO plus Jas enhanced the affinity of actin and GST-p53. In contrast,CD treatment led to less actin pulled down by GST-p53 in ETO treated cells (Fig. 3*B* *,* a and b). These results suggest that p53 preferentially interacts with polymeric actin, and that the p53-actin interaction is enhanced when DNA damage happens.

To further investigate whether the preferential interaction of p53 and polymeric actin exists in the cytoplasm or nucleus, YFP-p53 was transfected into U2OS cells because cytoplasmic p53 was too rare to be stained, and confocal-microscopy was performed. Phalloidin, specifically binding to F-actin, was used to detect the F-actin alternation. In control cells, F-actin appeared as parallel and thick stress fibers through cells (Fig. 3*C* a). YFP-p53 were distributed both in the cytoplasm and the nucleus (Fig. 3*C* b) and co-localized with actin fibers (Fig. 3*C* c). Jas treatment led to the formation of actin aggregates (Fig. 3*C* d). Punctate yellow fluorescence in the merged image showed the co-localization of actin and YFP-p53 (Fig. 3*C* f). F-actin signals decreased following CD treatment (Fig. 3*C* g), along with which, YFP-p53 tended to accumulate into the nucleus (Fig. 3*C* h). There was no obvious co-localization of actin and YFP-p53 either in the nucleus or in the cytoplasm (Fig. 3*C* i). Compared with normal cells, denser actin fibers formed in the ETO treated cells (Fig. 3*C* j), and YFP-p53 obviously accumulated in the nucleus in response to DNA damage (Fig. 3*C* k). Nevertheless, the co-localization of p53 and actin fibers mainly existed in the cytoplasm (Fig. 3*C* l). While Jas treatment resulted in p53 co-localizing with actin fibers and formation of aggregates (Fig. 3*C* o), CD treatment reduced their co-localization in ETO treated cells (Fig. 3*C* r). Then, the colocalization ratio of F-actin and YFP-p53 was analyzed with Image J (more than 100 cells were calculated) (Fig. 3D).

Together, the combined data suggest that there is a high affinity between polymeric actin and p53 in the cytoplasm, and this affinity is enhanced upon ETO treatment.

### Actin Polymerization Impairs p53 Nuclear Import

**Figure 4:**
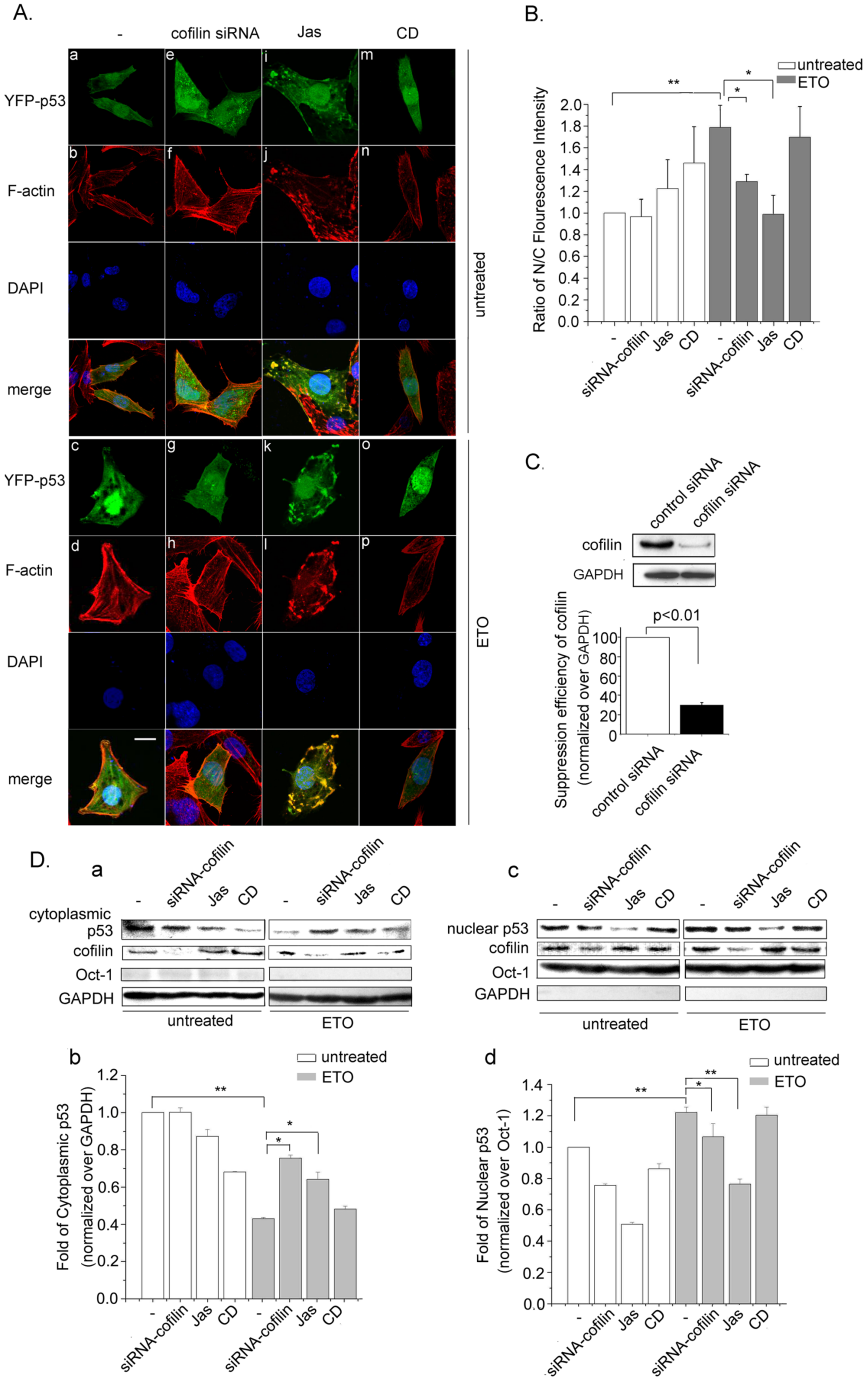
Actin polymerization impairs p53 nuclear import. *A.* Cells were transfected with siRNA-cofilin or treated with Jas (50 nM) or CD (0.01 µg/ml) for 2 h before YFP-p53 transfection. 24 h after YFP-p53 transfection, cells were treated with ETO (10 µM) or untreated for another 12 h. Cells were then analyzed by confocalmicroscopy. Scale bar, 10 µm. *B.* Fluorescence values of more than 100 cells were calculated using Image J software. Results are presented as means ± SD of values from three independent experiments. *C*. Cells were transfected with control siRNA or cofilin siRNA, whole cell protein extraction and western blotting were then carried out to detect cofilin suppression efficiency. Panels of western blotting were analyzed with Image J software. Results are presented as means ± SD of values from three independent experiments. *D.* Cells were transfected with siRNA-cofilin or treated with Jas (50 nM) or CD (0.01µg/ml) for 2 h before cells were treated with ETO (10 µM) or untreated as control for another 12 h. Cytoplasmic protein and nuclear proteins were then extracted, and western blotting was performed. Oct-1 (octamer transcription factor 1) was used as nuclear protein marker and GAPDH was used as cytoplasmic protein marker (a and c). Panels of western blotting were analyzed with Image J software. Results are presented as means ± SD of values from three independent experiments (b and d). All Statistical differences were determined by One-way ANOVA. *, P<0.05; **, P<0.01.

As p53 nuclear import is a key step of p53 accumulation, confocal microscopy was performed to detect p53 cellular localization. As shown in Fig. 4*A*, after transfection, ectopically expressed YFP-p53 was distributed in both the cytoplasm and the nucleus (Fig. 4*A*, a). ETO treatment resulted in the elevation of nuclear YFP-p53 (Fig. 4*A*, c). Cofilin is a small ubiquitous protein (∼19 kD) able to bind both monomeric (G-) and filamentous (F-) actin [36]. By severing actin filaments, cofilin increases the number of filament endings for polymerization and depolymerization [37], [38]. Thus, we performed cofilin siRNA transfection with the aim to stabilize cytoplasmic filamentous actin. Transfection of siRNA-cofilin resulted in the formation of F-actin aggregates (Fig. 4*A*, f and h), and impaired p53 nuclear accumulation (Fig. 4*A*, e), even in the ETO treated cells (Fig. 4*A*, g). Jas treatment induced YFP-p53 aggregates (Fig. 4*A*, i and k) along with actin masses formation in the cytoplasm (Fig. 4*A*, j and l), and inhibited p53 nuclear accumulation caused by ETO treatment (Fig. 4*A*, k). Less actin filaments were detected in the cells with CD treatment either in presence or absence of ETO (Fig. 4*A*, n and p), however, YFP-p53 remarkably accumulated in the nucleus of the cells treated with ETO (Fig. 4*A*, o) compared with that in the cells without ETO treatment (Fig. 4*A*, m). Then, the nuclear and the cytoplasmic fluorescence intensities of YFP-p53 were analyzed with Image J, and the ratio of nuclear/cytoplasmic fluorescence intensity was analyzed (Fig. 4*B*). The accumulation of nuclear p53 in ETO treated cells was almost two fold of control cells. There was no efficient nuclear import of p53 in cells that were transfected with cofilin siRNA followed by ETO treatment compared with single ETO treatment. Similar result was observed with Jas plus ETO treated cells. In contrast, CD treatment did not hamper p53 nuclear import. The suppression efficiency of cofilin siRNA was tested (Fig. 4*C*).

To further evaluate the effect of actin polymerization on p53 nuclear import, western blotting was employed to examine endogenous p53 levels in the cytoplasm and the nucleus respectively. Equivalent volume of protein extracts were separated by SDS-PAGE, and Image J was used to analyze the western blotting results. The fold change of nuclear and cytoplasmic p53 was calculated. The protein level of cytoplasmic p53 reduced to 40% of control by ETO treatment (Fig. 4*D* *,* a and b). Additionally, cofilin siRNA plus ETO treatment induced a 2-fold increase of p53 in the cytoplasm compared with ETO treatment only (Fig. 4*D* *,* a and b). In cells treated with Jas plus ETO, the quantity of cytoplasmic p53 was also obviously higher than that in the cells with ETO treatment alone. The results of CD plus ETO treatment and ETO treatment alone were similar (Fig. 4*D* *,* a and b). Then, the level of nuclear protein was determined. ETO treatment resulted in a significant increase of nuclear p53 compared to those without ETO treatment (Fig. 4*D* *,* c and d). Interestingly, even without ETO treatment, cofilin siRNA and Jas but not CD resulted in a clear reduction of nuclear p53. While, in cells with ETO treatment, cofilin siRNA transfection resisted the nuclear import of p53, and the nuclear p53 level was significantly lower than ETO treatment alone (Fig. 4*D* *,* c and d). Moreover, while the presence of Jas dramatically eliminated the enhanced nuclear accumulation of p53, there was no reduction of p53 nuclear import with CD treatment in ETO-treated cells (Fig. 4*D* *,* c and d). The results revealed from western blotting analysis were consistent with the results of confocal microscopy. The combined data demonstrate that nuclear import of p53 was impaired by actin polymerization.

### Actin Polymerization Affects the Ser315 Phosphorylation and the Ubiquitination of p53

**Figure 5:**
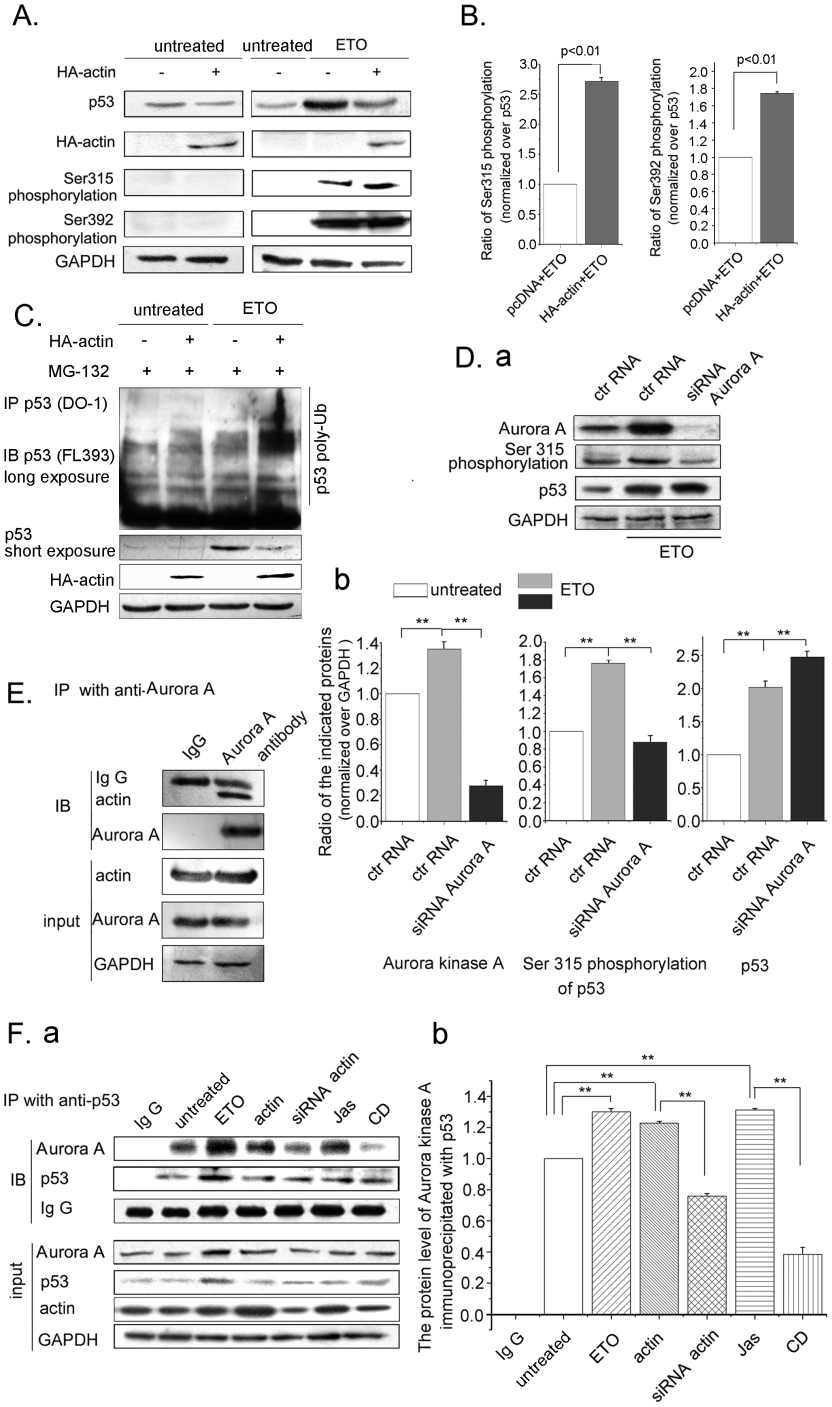
Actin polymerization affects the Ser315 phosphorylation and ubiquitination of p53. *A.* U2OS cells transfected with HA-actin were treated with ETO (10 µM) or untreated as control for 12 h. The cells were then harvested and whole cell proteins were extracted for western blottings with indicated antibodies. *B.* Western blottings were analyzed using Image J software. Results are presented as mean ± SD of values from three independent experiments. *C.* U2OS cells transfected with HA-actin. At 24 h after the transfection, cells were treated with or without ETO for 12 h, followed by the treatment of 20 µM MG132 for 6 h. Whole cell extracts were immunoprecipitated with anti-p53 (DO-1) antibody and analyzed by western blotting with anti-p53 (FL-393) antibody. *D*. Cells were transfected with Aurora kinase A siRNA, and 24 h after transfection, cells were treated with ETO (10 µM) or untreated as control for 12 h. Then, cells were harvested and whole cell proteins were extracted for western blotting (a). Western blottings were analyzed using Image J software. Results are presented as mean ± SD of values from three independent experiments (b). *E*. Cells were harvested and subjected to immunoprecipitations using anti-Aurora A antibody and analyzed by western blotting. *F*. Cells were transfected with actin or actin siRNA, or treated with ETO (10 µM, 12 hours) or untreated as control, or CD (0.01µg/ml) and Jas (50 nM) for 2 h. Cells were then harvested and subjected to immunoprecipitations using anti-p53 antibody and analyzed by western blotting (a). Western blotting results were analyzed using Image J software. Results are presented as mean ± SD of values from three independent experiments (b). All Statistical differences were determined by One-way ANOVA. **, P<0.01.

Since the polymeric actin, which forms in response to DNA damage, hampers p53 entering into the nucleus, we questioned the subsequent destination of these “rejected” p53 in the cytoplasm. Previous studies showed that phosphorylation at Ser392 stabilized the formation of p53 tetramer [39], [40]. In contrast, the phosphorylation of Ser315 reversed the activating effects of Ser392 phosphorylation on both tetramer formation and stabilization of p53 [41], [42]. Moreover, phosphorylation of p53 at Ser315 inactivated p53 by enhancing its proteolytic degradation [43], [44]. Therefore, we examined the phosphorylation levels of p53 at Ser315 and Ser392. Cells were transfected with HA-actin to enhance the level of actin. As shown in Fig. 5*A*, the protein level of p53 was less in the actin-transfected and ETO-treated cells than the cells treated with ETO only. Also, the phosphorylation at Ser315 of p53 was increased to 2.75 folds compared with the controls transfected with pcDNA vector, but only 1.7 folds was observed on the phosphorylation at Ser392 (Fig. 5*A* and Fig. 5*B*). Then, we analyzed the ubiquitination of p53 by using MG-132 to block the 26S proteasomal activity. Actin transfection led to an increase in ubiquitination of p53 (Fig. 5*C*). These results imply that the degradation of p53 is promoted in part by polymeric actin formation in response to DNA damage.

As Aurora kinase A participates in the phosphorylation of p53 at Ser315 [44], we next examined the ability of Aurora kinase A in the phosphorylation of p53 at Ser315. Transfection with Aurora kinase A siRNA resulted in an obvious decrease of Aurora kinase A expression (Fig. 5*D*, a and b). While the phosphorylation level of p53 at Ser315 decreased, there was a detectable increase of p53 in Aurora kinase A siRNA transfected cells (Fig. 5*D*, a and b). To investigate the connection between actin polymerization, Aurora kinase A and p53, co-immunoprecipitation was performed with Aurora kinase A antibody. As shown in Fig. 5*E*, actin associated with Aurora kinase A in the same complex. Then, the quantity of Aurora kinase A in the p53-associated complexes under different conditions was examined. As shown in Fig. 5*F*, ETO treatment enhanced p53 level and induced more Aurora kinase A interaction with p53. When cells were transfected with actin, increased Aurora kinase A was observed to interact with p53 compared with the control. In contrast, transfection with actin siRNA decreased the co-immunoprecipitation of p53 and Aurora kinase A. Likewise, Jas treatment resulted in more Aurora kinase A precipitating with p53, whereas, in the cells with CD treatment, lower level of precipitated Aurora kinase A was detected (Fig. 5*F*, a and b). These results suggest that actin polymerization promotes the interaction of Aurora kinase A and p53, which facilitates the phosphorylation of p53 at Ser315 and its subsequent degradation.

### The Impact of Actin Polymerization on p53 Leads to the Alteration of p21 Expression

**Figure 6:**
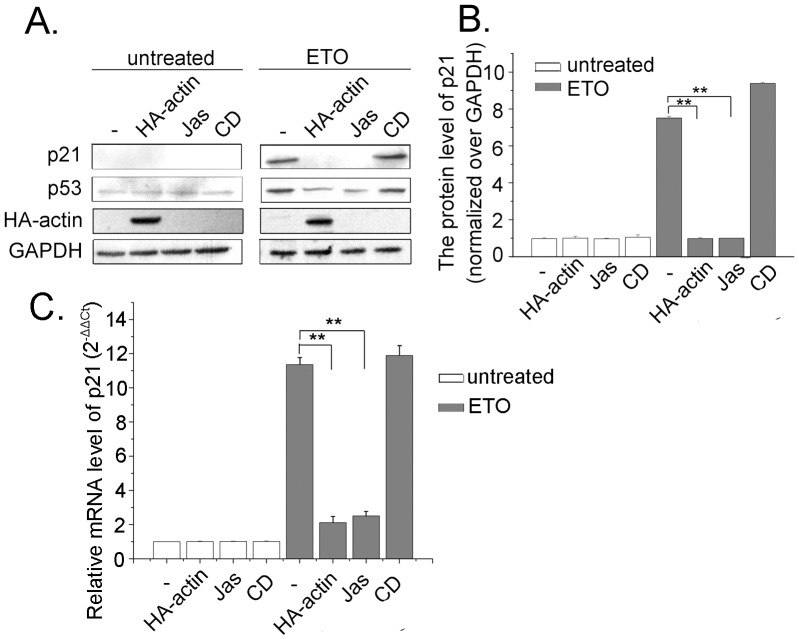
The impact of actin polymerization on p53 leads to the alteration of p21 expression. *A*. Cells were transfected with HA-actin or treated with Jas (50 nM) or CD (0.01 µg/ml) for 2 h. Then, cells were treated with ETO (10 µM) or untreated as control for 12 h. Whole cell proteins were extracted and western blotting was performed to measure p21 protein levels. *B.* Western blotting were analyzed with Image J software, and the results are presented as mean ± SD of values from three independent experiments. *C.* Cells were transfected with HA-actin or treated with Jas (50 nM) or CD (0.01 µg/ml) for 2 h. Then, cells were treated with ETO (10 µM) or untreated as control for 12 h. Real-Time PCR was performed, mRNA content of p21 was normalized to that of GAPDH and the normal cells’ mRNA level was valued as 1. Data (mean±SD) were from three independent experiments. All Statistical differences were determined by One-way ANOVA. **, P<0.01.

When DNA damage occurs, p53 is activated and binds to the promoters of p21 and/or other target genes, resulting in cell cycle arrest or apoptosis [45]. Thus, p21 has been used as a marker for p53 activity. Then, we investigated the effects of F-actin modulation on p53 target gene p21 to address the biological implication of the impact of actin polymerization on p53. HA-actin plasmids, Jas and CD were used to increase or decrease F-actin content in cells, respectively. As shown in Fig. *6A*, p53 kept on a very low level and p21 had no expression in untreated cells. In the cells treated with ETO, p53 had an increase and p21 expression was induced obviously. When cells were transfected with HA-actin or treated with Jas, less p53 was dectected. As a consequence, no obvious p21 was shown. Conversely, there was no decrease of p53 accumulation and p21 expression with CD treatment. Image J was used to analyze the western blotting results (Fig. *6B*). As a measure of p53 transcriptional activity, we looked into the levels of p21 mRNA, a well established p53 target gene. Transfection of HA-actin and Jas treatment effiently decreased p21 mRNA level in ETO treated cells (Fig. 6*C*). All the above data suggest that the effects of actin polymerization on p53 nuclear import and ubiquitination impact p53 transcriptional activity on its target gene.

## Discussion

Actin exists in both monomeric (globular or G-actin) and polymeric (filamentous or F-actin) forms [46]. Both of them have remarkable conformational flexibility and adopt various structural states in response to interaction with their partner molecules [19]. Numerous physiological and pathological stimuli promote the rearrangement of actin cytoskeleton, thereby modulating cellular mobility [47]. Previous studies reported that actin could interact with p53 [48], [49]. However, there was no evidence to address how the interaction of actin and p53 responds to DNA damage in the regulation of p53 functions. In our study, we show that there is a slow increase of cell length/width ratio at the initial stage of DNA damage (Fig. 1*A and 1B*). The results of quantitative analyses of the fluorescence intensity for F-actin and immunofluorescence also confirm the increase of actin polymerization upon ETO treatment (Fig. 1*C and 1D*). These results are similar with the previous reports demonstrating actin assembly in response to UV radiation [50]. As actin polymerized in the cells that responded to double-strand and single-strand cleavage of DNA induced by ETO [51] or DNA lesions induced by UV radiation [52], we presumed that the increase of actin polymerization exists widely in response to different types of DNA damage.

Under normal conditions, the p53 protein is kept as a labile and inactive protein. When cells are exposed to DNA damage and other stress, p53 accumulates in the nucleus and becomes active. Previous studies reported that actin affects p53 accumulation indirectly. F-actin could inhibit the kinase activity of c-Abl, which facilitates p53 accumulation in cells [53], [54]. In this study, we found that actin polymerization directly impairs p53 accumulation. Furthermore, our results demonstrate that polymeric actin interacts with p53 in vivo and in vitro (Fig. 3*A and 3B*). In addition, YFP tagged p53 colocalizes with actin aggregation or amorphous masses induced by Jas treatment (Fig. 3*C and 3D*). These results have provided evidence that p53 are recruited to polymeric actin. Next, we investigated the localization of p53 in actin polymerized cells. In the presence of Jas or cofilin siRNA, p53 accumulation induced by ETO treatment in the nucleus decreased compared with ETO treatment alone (Fig. 4). All these findings suggest that actin polymerization directly impairs p53 nuclear accumulation by interacting with p53 in the cytoplasm.

p53 protein decreased when cells were treated with actin assembly agent (Fig.2*F* *a*), so we wanted to know what happened to the rejected cytoplasmic p53. Previous studies reported that phosphorylation of p53 at Ser315 inactivates p53 by enhancing its proteolytic degradation in the cytoplasm [43], which is facilitated by Aurora kinase A [44]. In this study, we found that actin polymerization increases the phosphorylation of p53 at Ser315 (Fig. 5*A and 5B*). Moreover, the ubiquitination of p53 increased when actin was polymerized (Fig. 5*C*). Then, we identified that actin polymerization facilitates p53 interaction with Aurora kinase A (Fig. 5*D*-5*F*), suggesting that actin polymerization probably regulates the ubiquitination of p53 by recruiting Aurora kinase A. p21 is identified as an inhibitor of cyclin-dependent kinases, and a mediator of p53 function [55]. Therefore, we detected p21 mRNA levels as indications of p53 activity. Our results demonstrated that actin polymerization decreases the mRNA level of p21, emphasizing the biological implication of actin polymerization on p53 function in response to DNA damage.

In summary, our data delineate the regulatory mechanism of actin dynamics in modulating p53 function, namely, actin becomes polymerized and negatively regulates p53 function by interacting with it in the cytoplasm, and thus impairs p53 nuclear import in response to DNA damage. In addition, p53 cytoplasmic retention increases its opportunity to interact with Aurora kinase A, which facilitates phosphorylation of p53 at Ser315 and p53 subsequent ubiquitination. In normal conditions, p53 remains at a very low level. In response to DNA damage, lower level of p53 tends to favor cell growth arrest, whereas higher level of p53 triggers cell apoptosis [17]. We presume that high polymeric actin formation could slow down the p53 nuclear import, which might let cells to gain time to repair their DNA damage, thereby reducing the injury caused by the overreaction in response to DNA damage.

## References

[1] Meek DW (2009) Tumor suppression by p53: a role for the DNA damage response? Nat Rev Cancer 9: 714–723. 19730431 10.1038/nrc2716

[2] Bremner R, Balmain A (1990) Genetic changes in skin tumor progression: correlation between presence of a mutant ras gene and loss of heterozygosity on mouse chromosome 7. Cell 61: 407–417. 2185890 10.1016/0092-8674(90)90523-h

[3] Fearon ER, Vogelstein BA (1990) Genetic model for colorectal tumorigenesis. Cell 61: 759–767. 2188735 10.1016/0092-8674(90)90186-i

[4] Baker SJ, Markowitz S, Fearon ER, Willson JK (1990) Suppression of human colorectal carcinoma cell growth by wild-type p53. Science 249: 912–915. 2144057 10.1126/science.2144057

[5] Diller L, Kassel J, Nelson CE, Gryka MA, Litwak G, et al (1990) p53 functions as a cell cycle control protein in osteosarcomas. Mol Cell Biol 10: 5772–5781. 2233717 10.1128/mcb.10.11.5772-5781.1990 PMC361354

[6] Mercer WE, Shields MT, Amin M, Sauve GJ, Appella E, et al (1990) Negative growth regulation in a glioblastoma tumor cell line that conditionally expresses human wild-type p53. Proc Natl Acad Sci USA 7: 6166–6170. 10.1073/pnas.87.16.6166 PMC54493 2143581

[7] Martinez J, Georgoff I, Martinez J, Levine AJ (1991) Cellular localization and cell cycle regulation by a temperature-sensitive p53 protein. Genes Dev 5: 151–159. 1995413 10.1101/gad.5.2.151

[8] Shaulsky G, Ben-Ze’ev A, Rotter V (1990a) Subcellular distribution of the p53 protein during the cell cycle of Balb/c 3T3 cells. Oncogene 5: 1707–1711. 2267137

[9] Marine JC (2010) p53 stabilization: the importance of nuclear import. Cell Death Differ 17: 191–192. 20062067 10.1038/cdd.2009.183

[10] Kruse JP, Gu W (2009) Modes of p53 regulation. Cell 137: 609–622. 19450511 10.1016/j.cell.2009.04.050 PMC3737742

[11] Moll UM, Riou G, Levine AJ (1992) Two distinct mechanisms alter p53 in breast cancer: Mutation and nuclear exclusion. Proc Natl Acad Sci USA 89: 7262–7266. 1353891 10.1073/pnas.89.15.7262 PMC49686

[12] Moll UM, LaQuaglia M, Benard J, Riou G (1995) Wild-type p53 protein undergoes cytoplasmic sequestration in undifferentiated neuroblastomas but not in differentiated tumors. Proc Natl Acad Sci USA 92: 4407–4411. 7753819 10.1073/pnas.92.10.4407 PMC41953

[13] Bosari S, Viale G, Roncalli M, Graziani D, Borsani G, et al (1995) p53 gene mutations, p53 protein accumulation and compartmentalization in colorectal adenocarcinoma. Am J Pathol 147: 790–798. 7677190 PMC1870957

[14] Alarcon VD, Ronai Z (2002) p53–Mdm2–the affair that never ends. Carcinogenesis 23: 541–547. 11960904 10.1093/carcin/23.4.541

[15] Stommel JM, Marchenko ND, Jimenez GS, Moll UM, Hope TJ, et al (1999) A leucine-rich nuclear export signal in the p53 tetramerization domain: regulation of subcellular localization and p53 activity by NES masking. EMBO J 18: 1660–1672. 10075936 10.1093/emboj/18.6.1660 PMC1171253

[16] Wadhwa R, Takano S, Robert M, Yoshida A, Nomura H, et al (1998) Inactivation of Tumor Suppressor p53 by Mot-2, a hsp70 Family Member. J Biol Chem 273: 29586–29591. 9792667 10.1074/jbc.273.45.29586

[17] Aylon Y, Oren M (2007) Living with p53, dying of p53. Cell 130: 597–600. 17719538 10.1016/j.cell.2007.08.005

[18] Staiger CJ, Blanchoin L (2006) Actin dynamics: old friends with new stories. Curr Opin Plant Biol 9: 554–562. 17011229 10.1016/j.pbi.2006.09.013

[19] Olson EN, Nordheim A (2010) Linking actin dynamics and gene transcription to drive cellular motile functions. Nat Rev Mol Cell Biol 11: 353–365. 20414257 10.1038/nrm2890 PMC3073350

[20] Carlsson AE (2010) Actin dynamics: from nanoscale to microscale. Annu Rev Biophys 39: 91–110. 20462375 10.1146/annurev.biophys.093008.131207 PMC2967719

[21] Hild G, Bugyi B, Nyitrai M (2010) Conformational dynamics of actin: Effectors and implications for biological function. Cytoskeleton (Hoboken) 67: 609–629. 20672362 10.1002/cm.20473 PMC3038201

[22] Metcalfe S, Weeds A, Okorokov AL, Milner J, Cockman M, et al.. (1999) Wild-type p53 protein shows calcium-dependent binding to F-actin. Oncogene 18, 2351–2355. 10.1038/sj.onc.1202559 10327055

[23] Jiang M, Axe T, Holgate R, Rubbi CP, Okorokov AL, et al (2001) p53 binds the nuclear matrix in normal cells: binding involves the proline-rich domain of p53 and increases following genotoxic stress. Oncogene 20: 5449–5458. 11571642 10.1038/sj.onc.1204705

[24] Sidani M, Wessels D, Mouneimne G, Ghosh M, Goswami S, et al (2007) Cofilin determines the migration behavior and turning frequency of metastatic cancer cells. J Cell Biol 179: 777–791. 18025308 10.1083/jcb.200707009 PMC2080932

[25] Wang XX, Liu R, Jin SQ, Fan FY, Zhan QM (2006) Overexpression of Aurora-A kinase promotes tumor cell proliferation and inhibits apoptosis in esophageal squamous cell carcinoma cell line. Cell Research 16: 356–366. 16617331 10.1038/sj.cr.7310046

[26] Li JQ, Li QX, Xie CC, Zhou HM, Wang YQ, et al (2004) β-actin is required for mitochondria clustering and ROS generation in TNF-induced, caspase-independent cell death. J Cell Sci 117: 4673–4680. 15371523 10.1242/jcs.01339

[27] Lambert CM Ngoka (2008) Sample prep for proteomics of breast cancer: proteomics and gene ontology reveal dramatic differences in protein solubilization preferences of radioimmunoprecipitation assay and urea lysis buffers. Proteome Sci 6: 30. 18950484 10.1186/1477-5956-6-30 PMC2600628

[28] Ivan M, Haberberger T, Gervasi DC, Michelson KS, Gunzler V, et al (2002) Biochemical purification and pharmacological inhibition of a mammalian prolylhydroxylase acting on hypoxia-inducible factor. Proc. Natl. Acad. Sci. USA 99: 13459–13464. 10.1073/pnas.192342099 PMC129695 12351678

[29] Pan X, Zhao J, Zhang WN, Li HY, Mu R, et al (2009) Induction of SOX4 by DNA damage is critical for p53 stabilization and function. PNAS 106: 3788–3793. 19234109 10.1073/pnas.0810147106 PMC2656158

[30] Smart DJ, Halicka HD, Schmuck G, Traganos F, Darzynkiewicz Z, et al (2008) Assessment of DNA double-strand breaks and γH2AX induced by the topoisomerase II poisons etoposide and mitoxantrone. Mutat Res 641: 43–47. 18423498 10.1016/j.mrfmmm.2008.03.005 PMC2581813

[31] Zuchero JB, Belin B, Mullins RD (2012) Actin binding to WH2 domains regulates nuclear import of the multifunctional actin regulator JMY. Mol Biol Cell 23: 853–863. 22262458 10.1091/mbc.E11-12-0992 PMC3290644

[32] Waldman T, Kinzler KW, Vogelstein B (1995) p21 is necessary for the p53-mediated G1 arrest in human cancer cells. Cancer Res 55: 5187–5190. 7585571

[33] Asker C, Wiman KC, Selivanova G (1999) p53-induced apoptosis as a safeguard against cancer. Biochem Biophys Res Commun 265: 1–6. 10548481 10.1006/bbrc.1999.1446

[34] Bubb MR, Spector I, Beyer BB, Fosen KM (2000) Effects of Jasplakinolide on the Kinetics of Actin Polymerization. J Biol Chem 275: 5163–5170. 10671562 10.1074/jbc.275.7.5163

[35] Heptinstall JA, May H, Ratan JR, Glenn WL (1998) GPIIb-IIIa antagonists cause rapid disaggregation of platelets pre-treated with cytochalasin D. Evidence that the stability of platelet aggregates depends on normal cytoskeletal assembly. Platelets 9: 227–232. 16793707 10.1080/09537109876744

[36] Maciver SK, Hussey PJ (2002) The ADF/cofilin family: actin-remodeling proteins. Genome Biol 3: 3007–3012. 10.1186/gb-2002-3-5-reviews3007 PMC139363 12049672

[37] Ono S (2007) The mechanism of depolymerization and severing of actin filaments and its significance in cytoskeletal dynamics. Int Rev Cytol 258: 1–82. 17338919 10.1016/S0074-7696(07)58001-0

[38] Ichetovkin I, Grant W, Condeelis J (2002) Cofilin produces newly polymerized actin filaments that are preferred for dendritic nucleation by the Arp2/3 complex. Curr Biol 12: 79–84. 11790308 10.1016/s0960-9822(01)00629-7

[39] Sakaguchi K, Sakamoto H, Lewis MS, Anderson CW, Erickson JW, et al (1997) Phosphorylation of serine 392 stabilizes the tetramer formation of tumor suppressor protein p53. Biochemistry 36: 10117–10124. 9254608 10.1021/bi970759w

[40] Engel K, Kotlyarov A, Gaestel M (1998) Leptomycin B-sensitive nuclear export of MAPKAP kinase 2 is regulated by phosphorylation. EMBO J 17: 3363–3371. 9628873 10.1093/emboj/17.12.3363 PMC1170674

[41] Gillotin S, Yap D, Lu X (2010) Mutation at Ser392 specifically sensitizes mutant p53H175 to mdm2-mediated degradation. Cell Cycle 9: 1390–1398. 20234175 10.4161/cc.9.7.11253

[42] Yap DB, Hsieh JK, Zhong S, Heath V, Gusterson B, et al (2004) Ser392 phosphorylation regulates the oncogenic function of mutant p53. Cancer Res 64: 4749–4754. 15256442 10.1158/0008-5472.CAN-1305-2

[43] Fogal V, Hsieh JK, Royer C, Zhong S, Lu X (2005) Cell cycle-dependent nuclear E2F1 requires phosphorylation. EMBO J 24: 2768–2782. 16037820 10.1038/sj.emboj.7600735 PMC1182237

[44] Katayama H, Sasai K, Kawai H, Yuan ZM, Bondaruk J, et al (2004) Phosphorylation by aurora kinase A induces Mdm2-mediated destabilization and inhibition of p53. Nat Genet 36: 55–62. 14702041 10.1038/ng1279

[45] Waldman T, Kinzler KW, Vogelstein B (1995) p21 is necessary for the p53-mediated G_1_ arrest in human cancer cells. Cancer Res 55: 5187–5190. 7585571

[46] Stuven T, Hartmann E, Gorlich D (2003) Exportin 6: A novel nuclear export receptor that is specific for profilin.actin complexes. EMBO J 22: 5928–5940. 14592989 10.1093/emboj/cdg565 PMC275422

[47] Bugyi B, Carlier MF (2010) Control of actin filament treadmilling in cell motility. Ann Rev Biophys 39: 449–470. 20192778 10.1146/annurev-biophys-051309-103849

[48] Metcalfe S, Weeds A, Okorokov AL, Milner J, Cockman M, et al (1999) Wild-type p53 protein shows calcium-dependent binding to F-actin. Oncogene 18: 2351–2355. 10327055 10.1038/sj.onc.1202559

[49] Katsumoto T, Higaki K, Ohno K, Onodera K (1995) Cell-cycle dependent biosynthesis and localization of p53 protein in untransformed human cells. Biol Cell 84: 167–173. 8720437 10.1016/0248-4900(96)89426-3

[50] Zuchero JB, Belin B, Mullins RD (2012) Actin binding to WH2 domains regulates nuclear import of multifunctional actin regulator JMY. Mol Boil Cell 23: 853–863. 10.1091/mbc.E11-12-0992 PMC3290644 22262458

[51] Korwek Z, Sewastianik T, Bielak-Zmijewska A, Mosieniak G, Alster O (2012) Inhibition of ATM blocks the etoposide-induced DNA damage response and apoptosis of resting human T cells. DNA Repair 11: 864–873. 23058634 10.1016/j.dnarep.2012.08.006

[52] Rastogi RP, Richa Kumar A, Tyagi MB, Sinha RP (2010) Molecular mechanisms of ultraviolet radiation-induced DNA damage and repair. J Nucleic Acids 2010: 592980. 21209706 10.4061/2010/592980 PMC3010660

[53] Woodring PJ, Hunter T, Wang JY (2001) Inhibition of c-Abl tyrosine kinase activity by filamentous actin. J Biol Chem 276: 27104–27110. 11309382 10.1074/jbc.M100559200

[54] Sionov RV, Coen S, Goldberg Z, Berger M, Bercovich B, et al (2001) c-Abl regulates p53 levels under normal and stress conditions by preventing its nuclear export and ubiquitination. Mol Cell Boil 21: 5869–5878. 10.1128/MCB.21.17.5869-5878.2001 PMC87306 11486026

[55] Jung YS, Qian Y, Chen X (2010) Examination of the expanding pathways for the regulation of p21 expression and activity. Cell Signal 22: 1003–1012. 20100570 10.1016/j.cellsig.2010.01.013 PMC2860671

